# Supplementary material for: Mitochondrial metabolism sustains CD8+ T cell migration for an efficient infiltration into solid tumors
Source: Nat Commun. 2024 Mar 11;15:2203. doi: 10.1038/s41467-024-46377-7 (PMC10928223; doi:10.1038/s41467-024-46377-7)
Supplement: Supplementary file 1 — Supplementary Information [file 41467_2024_46377_MOESM1_ESM.pdf]

Mitochondrial metabolism sustains $CD8^+$ T cell migration for an efficient infiltration into solid tumors

Luca Simula[1,*], Mattia Fumagalli[1], Lene Vimeux[1], Irena Rajnpreht[1], Philippe Icard[2,3], Gary Birsen[4], Dongjie An[1], Frédéric Pendino[1], Adrien Rouault[1], Nadège Bercovici[1], Diane Damotte[5], Audrey Lupo-Mansuet[5], Marco Alifano[3,6], Marie-Clotilde Alves-Guerra[7], and Emmanuel Donnadieu[1,*]

[1]*Institut Cochin, Inserm U1016, CNRS UMR8104, Université de Paris-Cité, Paris, 75014, France. Equipe labellisée "Ligue contre le Cancer"*

[2]*Université de Normandie, UNICAEN, Inserm U1086 Interdisciplinary Research Unit for Cancer Prevention and Treatment, Caen, France*

[3]*Thoracic Surgery Department, Cochin Hospital, APHP-Centre, Université Paris-Descartes, Paris, France*

[4]*Department of Pneumology, Thoracic Oncology Unit, Cochin Hospital, APHP-Centre, Université Paris-Cité, 75014 Paris, France*

[5]*Department of Pathology, Cochin Hospital, APHP-Centre, Université Paris-Cité, 75014 Paris, France.*

[6]*Inserm U1138, Integrative Cancer Immunology Unit, 75006 Paris, France*

[7]*Institut Cochin, Inserm U1016, CNRS UMR8104, Université de Paris-Cité, Paris, 75014, France.*

[*]correspondence: luca.simula@inserm.fr; emmanuel.donnadieu@inserm.fr

SUPPLEMENTARY INFORMATION

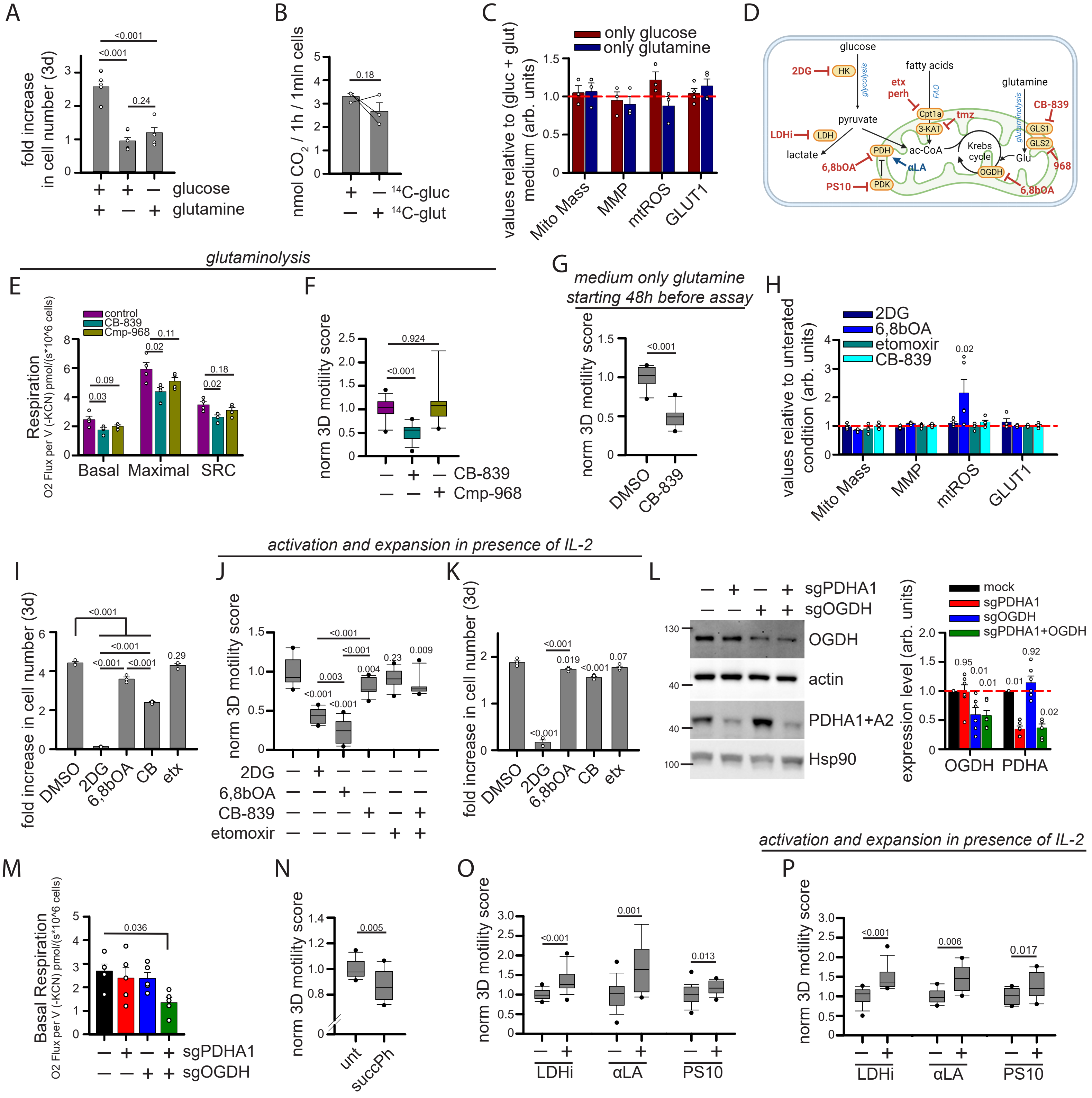

Supplementary Figure 1. Further analyses about the metabolic regulation of T cell 3D motility. Related to Figure 1.
(**A**) Fold increase in cell number of activated $CD8^+$ T cells cultured for 3d in absence of glucose or glutamine (n=7, indep. biological samples, One-Way ANOVA posthoc Holm Sidak's). (**B**) CO_2 production from radio-labelled glucose or glutamine in activated $CD8^+$ T cells (n=3 indep. biological samples, unpaired Student's T-Test). (**C, H**) Mitochondrial mass (Mito Mass), membrane potential (MMP), mtROS and GLUT1 expression in activated $CD8^+$ T cells in medium containing glutamine and/or glucose (C, n=3 indep. biological samples [n=4 for GLUT1], One-Way ANOVA or ANOVA on Ranks) or in presence of the indicated drugs (H, n=4 indep. biological samples [n=5 for mtROS], One-Way ANOVA or ANOVA on Ranks) compared to control condition (set as 1). (**D**) Scheme of drugs used to inhibit or activate metabolic enzymes and regulatory proteins. (**E-F**) Mitochondrial respiration (E, n=4 indep. biological samples, One-Way ANOVA posthoc Holm Sidak's) and motility in collagen gel (F, n=12 movies, ANOVA on Ranks posthos Dunnet's vs ctrl) in presence of the indicated drugs. (**G**) Normalized 3D motility Score in presence of CB-839 of activated $CD8^+$ T cells cultured since 48h before assay in medium containing glutamine and no glucose (n=10 movies, unpaired Student's T-Test). (**I**) Fold increase in cell number of activated $CD8^+$ T cells cultured for 3d in presence of the indicated drugs (n=3 indep. biological samples, One-Way ANOVA posthoc Holm Sidak's). (**J-K**) Normalized 3D motility Score (J, n=11 movies, One-Way ANOVA posthoc Holm Sidak's) and fold increase in cell number after 3d of culture (K, n=4 indep. biological samples, One-Way ANOVA posthoc Holm Sidak's) in presence of the indicated drugs for activated $CD8^+$ T cells cultured in presence of IL-2. (**L**) WB analysis of protein expression in activated $CD8^+$ T cells CRISPR/Cas9-edited for the indicated genes (n=6 indep. biological samples, ANOVA on Rank posthoc Dunnet's). (**M**) Basal respiration in activated $CD8^+$ T cells CRISPR/Cas9-edited for the indicated genes (n=5 indep. biological samples, One-Way ANOVA on Rep. Meas. posthoc Holm Sidak's). Related to Figure 1J. (**N**) Normalized 3D motility Score of activated $CD8^+$ T cells in presence of OGDH inhibitor succinyl-phosphonate (succPh) (n=12 movies, unpaired Student's T-Test). (**O-P**) Normalized 3D motility Score of activated $CD8^+$ T cells in presence of the indicated drugs. Since initial activation, cells were cultured with IL7+IL15 (O, LDHi n=18, αLA n=15, PS10 n=21 movies, Rank Sum Test or unpaired Student's T-Test) or IL2 (P, LDHi & PS10 n=13, αLA n=9 movies Rank Sum Test or unpaired Student's T-Test). Data are shown as mean ± SEM in A-E, H, I and K-M, and as box plot (center line, median; box limits, upper and lower quartiles; whiskers, 1.5x interquartile range; points, 5th and 95th percentiles) in F, G, J, and N-P. Scheme in D has been created with BioRender.com.

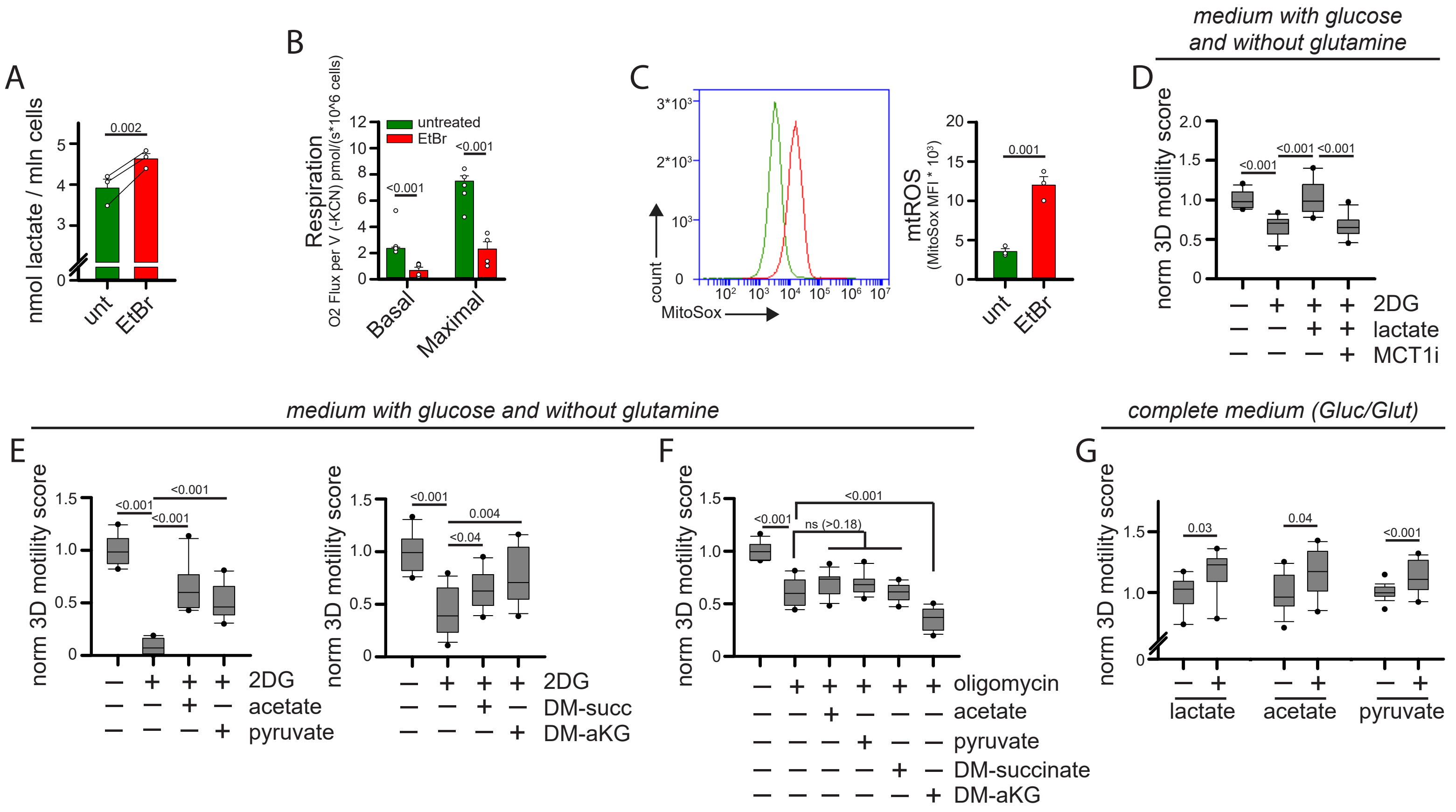

Supplementary Figure 2. Dissecting glycolysis/OXPHOS importance to sustain T cell 3D motility. Related to Figure 2.
(**A-C**) Activated CD8⁺ T cells were cultured in presence of ethidium bromide (EtBr) for at least 14d. Then, the amount of lactate (A, n=3, indep. biological samples, paired Student's T-Test), the basal and maximal respiration (B, n=4 indep. biological samples, unpaired Student's T-Test) and the amount of mtROS (MitoSox/FSC ratio; C, n=3 indep. biological samples, unpaired Student's T-Test) have been evaluated. (**D-F**) Normalized 3D motility Score of activated CD8⁺ T cells in presence of the indicated drugs (MCT1i, *i.e.*, AZD3965 in D, n=12 movies, One-Way ANOVA posthoc Holm Sidak's; 2DG in E, n=10 movies left graph, ANOVA on Ranks posthoc Tukey's, n=12 movies right graph, One-Way ANOVA posthoc Holm Sidak's; oligomycin in F, n=12 movies, One-Way ANOVA posthoc Holm Sidak's) and nutrients (dimethyl-succinate: DM-succ; dimethyl-alpha-keto-glutarate: DM-aKG) in a motility medium containing only glucose and no glutamine. Please note that data from columns 1-2 of figure S2F were also used in figure 2I for lactate since performed in a single whole experiment (data were splitted to improve quality of the presentation). (**G**) Normalized 3D motility Score of activated CD8⁺ T cells in presence of the indicated nutrients in a complete motility medium (glucose + glutamine) (lactate n=9, acetate n=12, pyruvate n=19 movies, unpaired Student's T-Test). Data are shown as mean ± SEM in A-C, and as box plot (center line, median; box limits, upper and lower quartiles; whiskers, 1.5x interquartile range; points, 5th and 95th percentiles) in D-G.

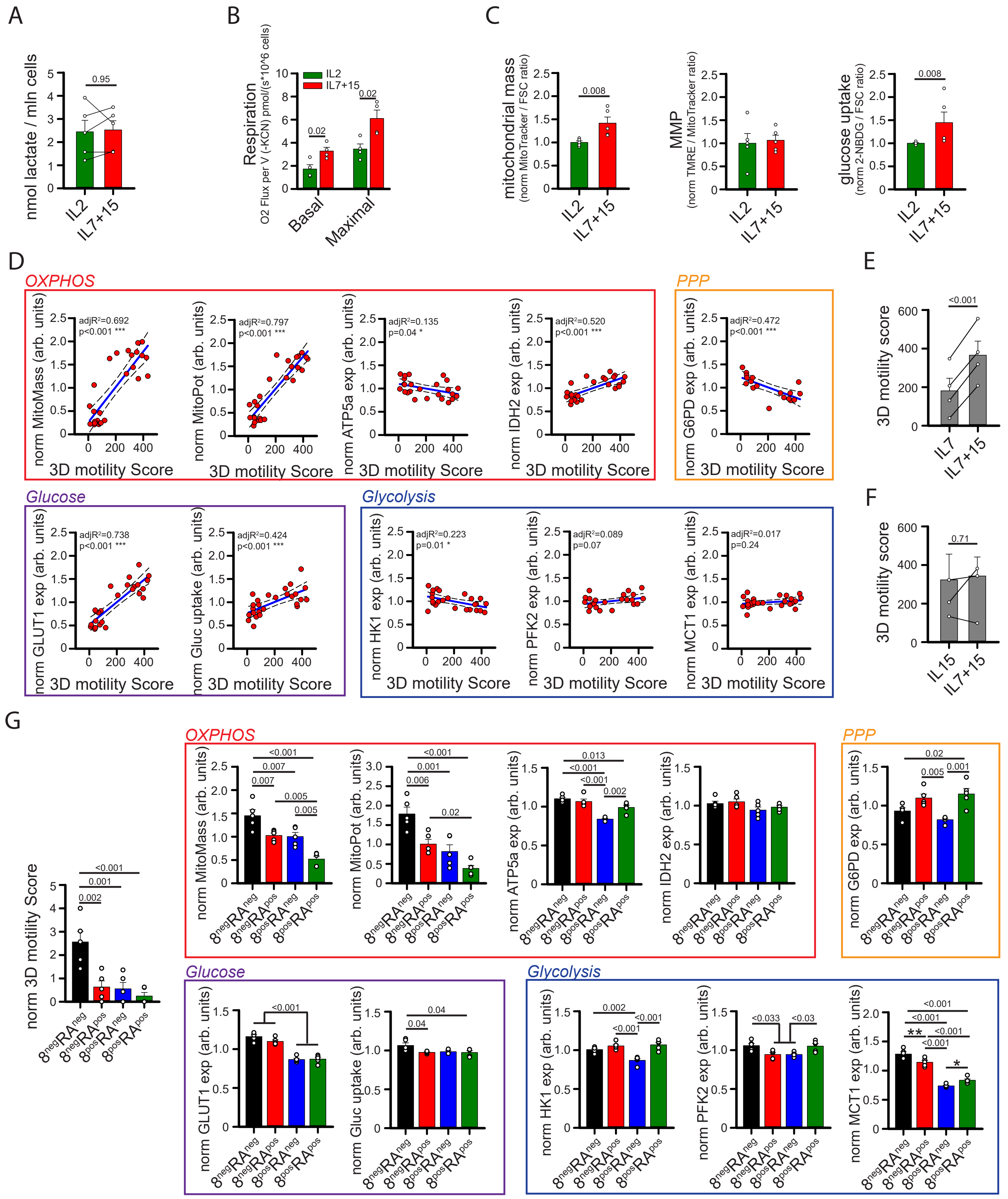

Supplementary Figure 3. Further correlations between T cell motility and mitochondrial metabolism. Related to Figure 3.
(**A-C**) CD8⁺ T cells have been activated and cultured in presence of IL2 or IL7+IL15 (indicated as IL7+15) (n=7). Then, the amount of lactate (A, n=5 indep. biological samples, paired Student‘s T-Test), the basal and maximal respiration (B, n=4 indep. biological samples, unpaired Student‘s T-Test), the amount of mitochondria (MitoTrackerGreen/FSC ratio C, n=5 indep. biological samples, Rank Sum Test), the mitochondrial membrane potential (MMP, TMRE/MitoTrackerGreen ratio; C, n=5 indep. biological samples, Rank Sum Test) and the amount of mtROS (MitoSox/FSC ratio; C, n=5 indep. biological samples, Rank Sum Test) have been evaluated. (**D**) CD8⁺ T cells were activated and cultured in presence of varying doses of IL7+15, IL2, and a-CD3/28 Abs. After 1 week, 3D motility and the level (MFI, flow cytometry) of indicated proteins or parameters (MitoMass: MitoTrackerGreen/FSC ratio; MitoPot: TMRE/MitoTrackerGreen ratio; Gluc uptake: 2-NBDG/FSC ratio) were evaluated. The graphs show the correlation between the normalized 3D motility Score and the indicated parameters (n=18 for G6PD, n=25 for ATP5a, GLUT1, IDH2 and HK1, n=26 samples for others, Linear Regression Test). (**E-F**) CD8⁺ T cells were activated and cultured in presence of IL7, IL15 or IL7+15. Then, cells were deposed onto tumor slice derived from the BxPC3-NSG model and their motility was evaluated as in Figure 3C (E, n=4 and F, n=3 slices, paired Student‘s T-Test). (**G**) 3D motility and the level (MFI) of the indicated proteins or parameters (as in D) were evaluated in freshly isolated hPBT cells not stimulated and stained with CD8 and CD45RA Abs to distinghuish the indicated populations (n=5 indep. biological samples, One-Way ANOVA posthoc Holm Sidak‘s). Data are shown as mean ± SEM in A-C, and E-G and as scatter plot including linear regression (mean ± 95% confidence interval) in D.

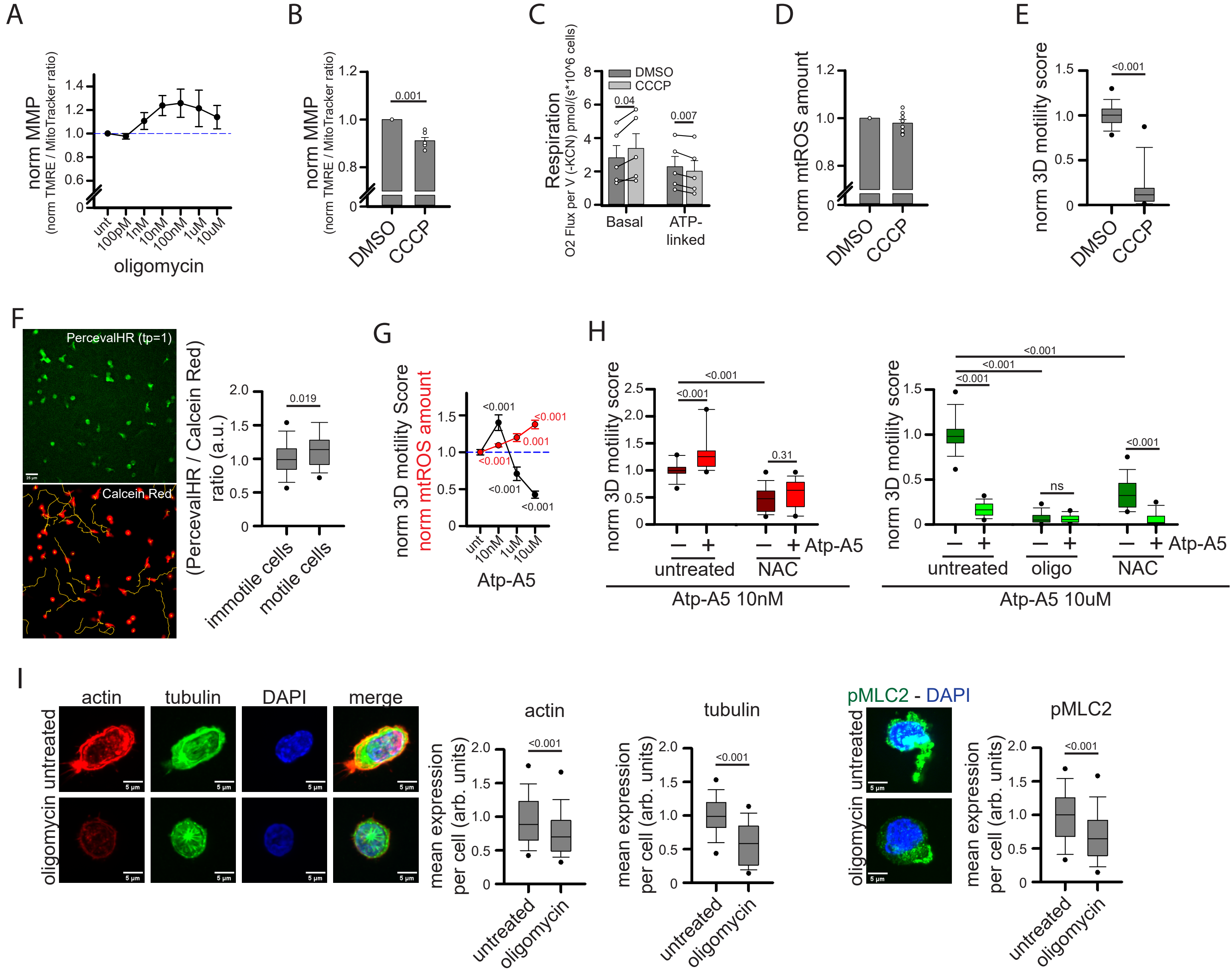

Supplementary Figure 4. Further analyses about the role of ATP and mtROS to sustain 3D motility. Related to Figure 4. (**A**) Normalized mitochondrial membrane potential (MMP, TMRE/MitoTrackerGreen ratio) in activated CD8$^+$ T cells cultured for 1h in presence of the indicated doses of oligomycin (n=5 indep. biological samples, ANOVA on Ranks posthoc Tukey's). (**B-E**) Activated CD8$^+$ T cells were cultured in presence of CCCP for 1h. Then, normalized mitochondrial membrane potential (MMP, TMRE/MitoTrackerGreen ratio; B, n=6 indep. biological samples, unpaired Student's T-Test), basal and ATP-linked respiration (C n=5 indep. biological samples, unpaired Student's T-Test), mtROS amount (Mitosox/FSC ratio; D, n=8 indep. biological samples, unpaired Student's T-Test) and normalized 3D motility Score (E, n=15 movies, unpaired Student's T-Test) were evaluated. (**F**) Activated CD8$^+$ T cells have been infected with lentiviral particles to express the ATP sensor PercevalHR. After 1w, cells were stained with Calcein Red and 3D motility assessed in collagen gel. PercevalHR MFI was calculated for each cell at first timepoint (tp=1) and then motility evaluated (tracks in yellow). Cells were distinghuished into immotile and motile cells according to their displacement and the relative [PercevalHR/CalceinRed] ratio was calculated as a proxy to infer ATP amount (n=62 immotile cells and n=45 motile cells, Rank Sum Test). (**G**) Normalized 3D motility Score (n=15 movies, ANOVA on Ranks posthoc Student-Newman-Keuls's) and mtROS amount (Mitosox/FSC ratio; n=6 indep. biological samples, ANOVA on Ranks posthoc Student-Newman-Keuls's) were evaluated in activated CD8$^+$ T cells in presence of the indicated doses of the ETC-II complex inhibitor Atpenin-A5 (Atp-A5). (**H**) Normalized 3D motility Score of activated CD8$^+$ T cells evaluated in presence of the indicated doses of Atpenin-A5 (Atp-A5), N-acetyl-cysteine (NAC) and oligomycin (oligo) (n=18 movies, ANOVA on Ranks posthoc Student-Newman-Keuls's). (**I**) Immunofluorescence analysis of the indicated proteins (pMLC2 = myosin light chain 2 phospho-Ser19) in activated CD8$^+$ T cells migrating into a collagen gel and treated or not with oligomyin. The quantification of the mean expression per cell is reported in the corresponding graphs (actin/tubulin, n=291 cells untreated, n=263 cells oligomycin from 4 blood donors; pMLC2, n=183 cells untreated, n=173 cells oligomycin from 3 blood donors, Rank Sum Test). Data are shown as mean ± SEM in A-D and G, and as box plot (center line, median; box limits, upper and lower quartiles; whiskers, 1.5x interquartile range; points, 5th and 95th percentiles) in E, F, H and I.

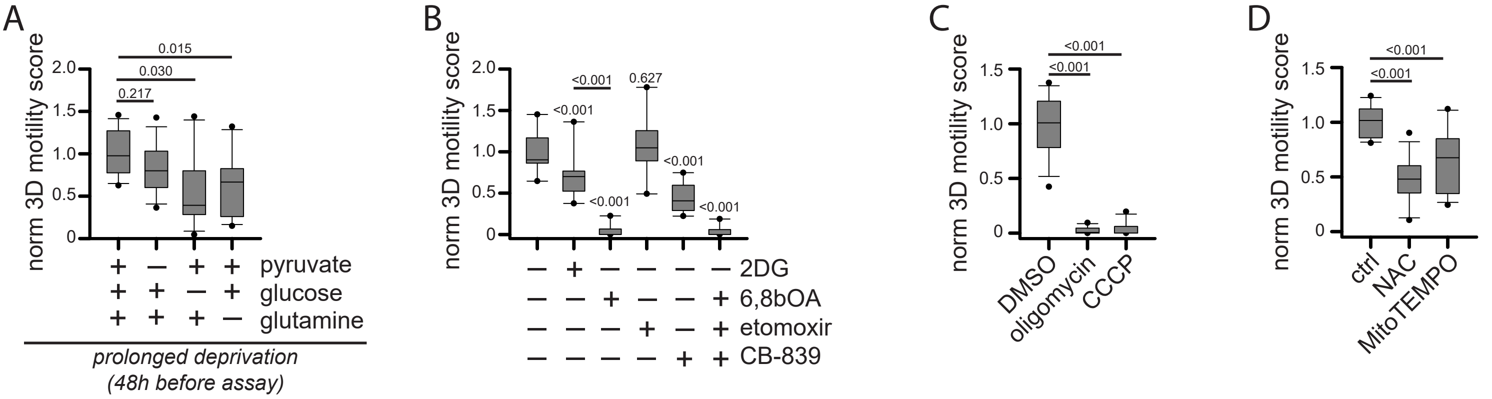

Supplementary Figure 5. Mitochondrial metabolism support CD4+ T cell 3D motility.

CD4+ T cells have been activated and expanded like CD8+ T cells (IL7+15). (**A**) Normalized 3D motility Score of activated CD4+ T cells cultured from 48h before assay in control medium or medium containing no glucose, no pyruvate or no glutamine (n=12 movies, One-Way ANOVA postoc Holm Sidak's). (**B-D**) Normalized 3D motility Score of activated CD4+ T cells in presence of the indicated drugs (B, n=9 movies, ANOVA on Ranks posthoc Student-Newman-Keuls's; C, n=12 movies, ANOVA on Ranks posthoc Dunnet's vs ctrl; D, n=12 movies, ANOVA on Ranks posthoc Holm Sidak's). All data are shown as box plot (center line, median; box limits, upper and lower quartiles; whiskers, 1.5x interquartile range; points, 5th and 95th percentiles).

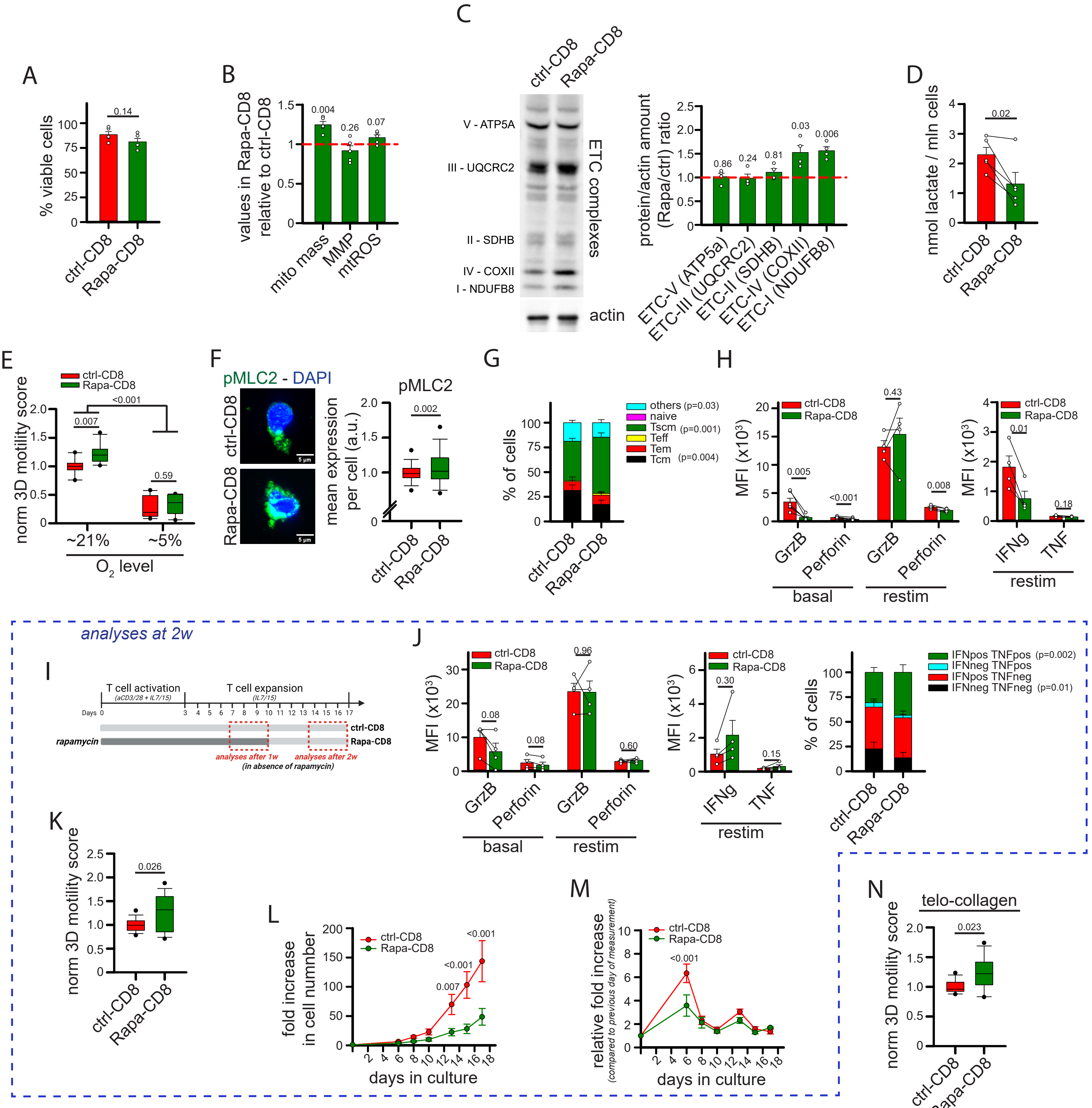

Supplementary Figure 6. Further analyses in rapamycin-treated CD8⁺ T cells. Related to Figure 5.
(**A-H**) Activated CD8⁺ T cells have been cultured in ctrl condition (ctrl-CD8) or in presence of rapamycin (Rapa-CD8). After 1w, rapamycin was removed the day of the analysis and the following parameters were measured: percentage of viable cells (A, n=5 indep. biological samples, unpaired Student's T-Test), the amount of mitochondria (mito mass: MitoTrackerGreen/FSC ratio in Rapa-CD8 relative to ctrl-CD8; B, n=5 indep. biological samples, unpaired Student's T-Test), the mitochondrial membrane potential (MMP: TMRE/MitoTrackerGreen ratio in Rapa-CD8 relative to ctrl-CD8; B, n=5 indep. biological samples, unpaired Student's T-Test), the amount of mtROS (MitoSox/FSC ratio in Rapa-CD8 relative to ctrl-CD8; B, n=5 indep. biological samples, unpaired Student's T-Test), the expression of the indicated proteins in Rapa-CD8 relative to ctrl-CD8 (WB, C, n=4 indep. biological samples, unpaired Student's T-Test), lactate production (D, n=5 indep. biological samples, unpaired Student's T-Test), the motility in collagen gel under different oxygen levels (E, n=10 movies, Two-Way ANOVA posthoc Tukey's), the relative proportion of indicated subsets (flow cytometry, G, n=6 indep. biological samples, unpaired Student's T-Test), and the level (MFI) of the indicated protein in resting (basal) or restimulated (restim, 5h with human TransAct + BfdA) cells (H, n=6 for basal, n=4 for restim indep. biological samples, unpaired Student's T-Test). In F, it is reported the immunofluorescence analysis of myosin light chain 2 phospho-Ser19 (pMLC2) expression in ctrl-CD8 and Rapa-CD8 migrating into a collagen gel. The relative mean pMLC2 expression per cell is reported in the graph on the right (n=310 cells ctrl-CD8, n=460 cells Rapa-CD8 from 3 donors, Rank Sum Test). (**I-M**) Activated CD8⁺ T cells have been cultured in control condition (ctrl-CD8) or in presence of rapamycin (Rapa-CD8) for 10d. Then, rapamycin was removed and cells were expanded in control conditions for additional 7d (I, scheme). The following analyses was performed at 14-17d: the level (MFI) of the indicated protein in resting cells (basal) or in cells restimulated (restim) 5h with human TransAct + BfdA (J, for IFNg and TNF the percentage of cells producing these cytokines is indicated in the graph on the right; n=4 indep. biological samples, unpaired Student's T-Test), the normalized 3D motility Score (K, n=27 movies, Rank Sum Test), the fold increase in cell number (L), and the relative fold increase in cell number (M) calculated as the fold increase at day "x" compared to previous day of measurement (L and M, n=5 indep. biological samples, Two-Way ANOVA on Rep. Measurements posthoc Holm Sidak's). (**N**) Normalized 3D motility Score of activated CD8⁺ T cells in 3mg/ml telocollagen gel (n=12 movies, Rank Sum Test). Data are shown as mean ± SEM in A-D, G-J, L and M, and as box plot (center line, median; box limits, upper and lower quartiles; whiskers, 1.5x interquartile range; points, 5th and 95th percentiles) in E, F, K and N. Scheme in I has been created with BioRender.com.

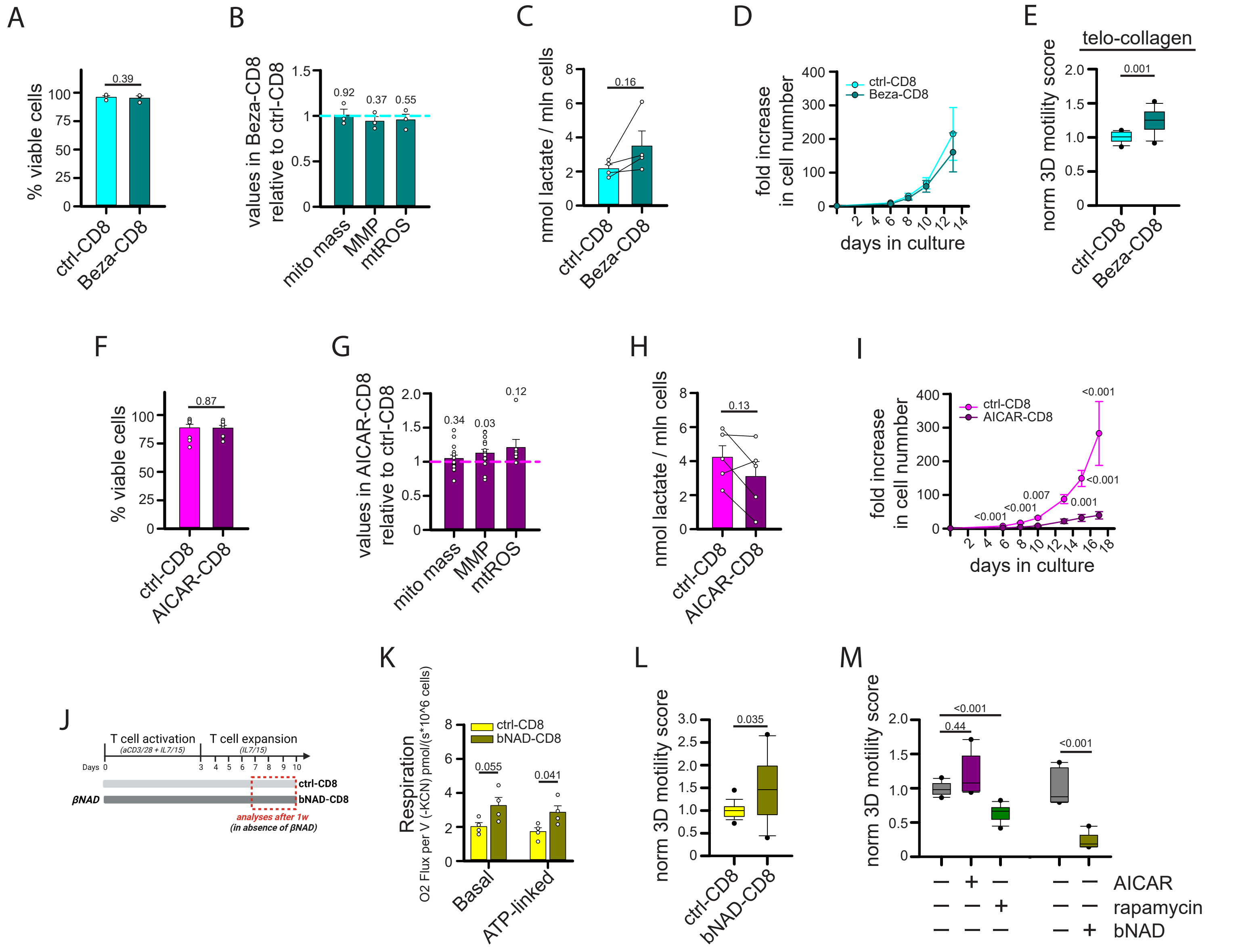

Supplementary Figure 7. Further analyses in bezafibrate- and AICAR-treated CD8$^+$ T cells. Related to Figure 5.
(**A-C**) Activated CD8$^+$ T cells have been cultured in ctrl condition (ctrl-CD8) or in presence of bezafirbate (Beza-CD8). After 1w, bezafibrate was removed the day of the analysis and the following parameters were measured: percentage of viable cells (A, n=3 indep. biological samples, unpaired Student's T-Test), the amount of mitochondria (mito mass: MitoTrackerGreen/FSC ratio in Beza-CD8 relative to ctrl-CD8; B, n=3 indep. biological samples, unpaired Student's T-Test), the mitochondrial membrane potential (MMP: TMRE/MitoTrackerGreen ratio in Beza-CD8 relative to ctrl-CD8; B, n=3 indep. biological samples, unpaired Student's T-Test), the amount of mtROS (MitoSox/FSC ratio in Beza-CD8 relative to ctrl-CD8; B, n=3 indep. biological samples, unpaired Student's T-Test), and lactate production (C, n=4 indep. biological samples, unpaired Student's T-Test). (**D**) Fold increase in cell number for ctrl-CD8 and Beza-CD8 T cells (obtained as in A). Bezafibrate was removed at 10d (n=4 indep. biological samples, ANOVA on Ranks posthoc Tukey's). (**E**) Normalized 3D motility Score of ctrl-CD8 and Beza-CD8 T cells (obtained as in A) in 3mg/ml telocollagen gel (n=12 movies, Rank Sum Test). (**F-H**) Activated CD8$^+$ T cells have been cultured in ctrl condition (ctrl-CD8) or in presence of AICAR (AICAR-CD8). After 3d, AICAR was removed and the cells expanded in control condition. The following parameters were measured starting from 7d: percentage of viable cells (F, n=9 indep. biological samples, unpaired Student's T-Test), the amount of mitochondria (mito mass: MitoTrackerGreen/FSC ratio in AICAR-CD8 relative to ctrl-CD8; G, n=15 indep. biological samples, unpaired Student's T-Test), the mitochondrial membrane potential (MMP: TMRE/MitoTrackerGreen ratio in AICAR-CD8 relative to ctrl-CD8; G, n=15 indep. biological samples, unpaired Student's T-Test), the amount of mtROS (MitoSox/FSC ratio in AICAR-CD8 relative to ctrl-CD8; G, n=7 indep. biological samples, unpaired Student's T-Test), and lactate production (H, n=5 indep. biological samples, unpaired Student's T-Test). (**I**) Fold increase in cell number for ctrl-CD8 and AICAR-CD8 T cells (obtained as in F). AICAR was removed at 3d (n=4 indep. biological samples, ANOVA on Ranks posthoc Student-Newman-Keuls's). (**J-L**) CD8$^+$ T cells have been cultured in ctrl condition (ctrl-CD8) or in presence of βNAD (bNAD-CD8). After 1w, βNAD was removed the day of the analysis (J, scheme) and the following parameters were measured: basal and ATP-linked respiration (K, n=4 indep. biological samples, unpaired Student's T-Test) and normalized 3D motility Score (L, n=18 movies, Rank Sum Test). (**M**) Normalized 3D motility Score of untreated activated CD8$^+$ T cells in presence of the indicated drugs added only during the assay (AICAR & Rapa, n=12 movies, ANOVA on ranks posthoc Dunnet's vs ctrl; bNAD, n=6 movies, unpaires Student's T-test). Data are shown as mean ± SEM in A-D and F-K, and as box plot (center line, median; box limits, upper and lower quartiles; whiskers, 1.5x interquartile range; points, 5th and 95th percentiles) in E, L and M. Scheme in J has been created with BioRender.com.

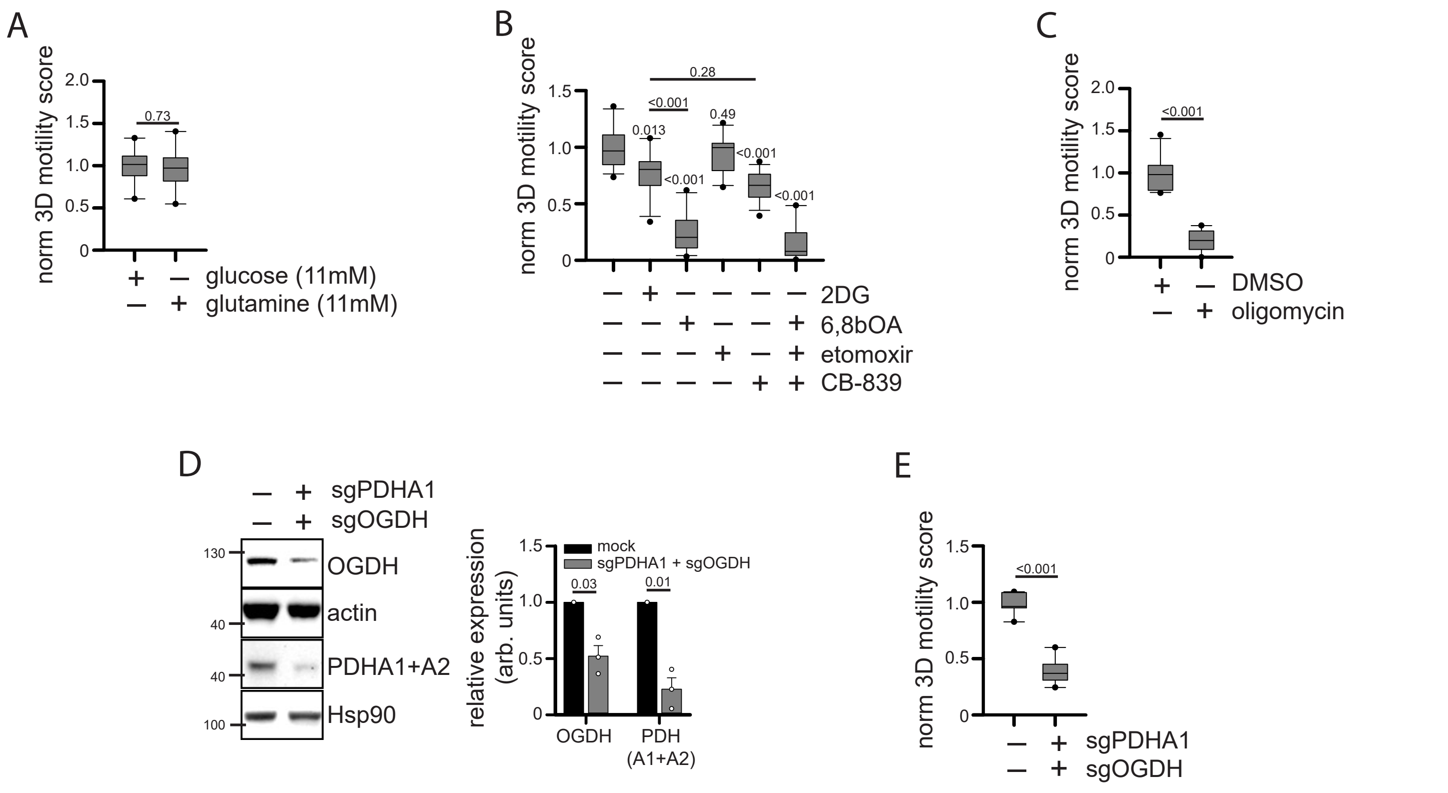

Supplementary Figure 8. Mitochondrial metabolism supports anti-EGFR CD8+ CAR T cell 3D motility.
(**A-C**) Activated CD8+ T cells have been infected with lentiviral particles to generate CD8+ CAR T cells recognizing the EGFR antigen (anti-EGFR CD8+ CAR T cells). After 1w, normalized 3D motility Score was calculated in motility medium containing no glucose or no glutamine (A, n=8 movies, unpaired Student's T-Test) or in the presence of the indicated drugs (B, n=12 movies, One-Way ANOVA posthoc Holm Sidak's; C, n=12 movies, unpaired Student's T-Test). (**D-E**) Anti-EGFR CD8+ CAR T cells (obtained as in A) were CRISPR/Cas9-edited for the indicated genes. After 1w, the levels of the indicated protein (WB analysis; D, n=3 indep. biological samples, unpaired Student's T-Test) and the normalized 3D motility Score (E, n=9 indep. biological samples, unpaired Student's T-Test) were evaluated. Data are shown as mean ± SEM in D, and as box plot (center line, median; box limits, upper and lower quartiles; whiskers, 1.5x interquartile range; points, 5th and 95th percentiles) in A-C and E.

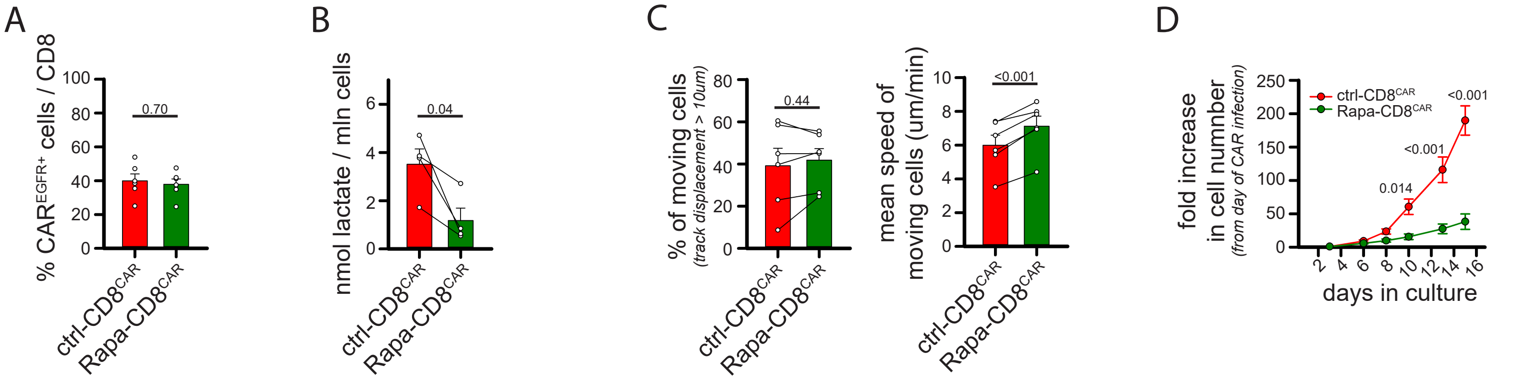

Supplementary Figure 9. Mitochondrial metabolism supports anti-EGFR $CD8^+$ CAR T cell 3D motility. Related to Figure 6.
(**A-C**) Activated $CD8^+$ T cells have been infected with lentiviral particles to generate $CD8^+$ CAR T cells recognizing the EGFR antigen (anti-EGFR $CD8^+$ CAR T cells) and cultured in control conditions (ctrl-$CD8^{CAR}$) or in presence of rapamycin (Rapa-$CD8^{CAR}$). After 1w, rapamycin was removed the day of the analysis and the following parameters were measured: percentage of cells expressing the anti-EGFR CAR molecule at the surface (A, n=6 indep. biological samples, unpaired Student's T-Test), lactate amount (B, n=4 indep. biological samples, unpaired Student's T-Test), and the two parameters composing the 3D motiity Score calculated in Fig. 6C: the percentage of moving cells and the mean speed of moving cells calculated as mean values for each donor (C, n=6 indep. biological samples, unpaired Student's T-Test).
(**D**) Fold increase in cell number for ctrl-$CD8^{CAR}$ and Rapa-$CD8^{CAR}$ T cells (obtained as in A). Rapa was removed at 10d (n=4 indep. biological samples, ANOVA Two-Way on Repeated Measurements). All data are shown as mean ± SEM.

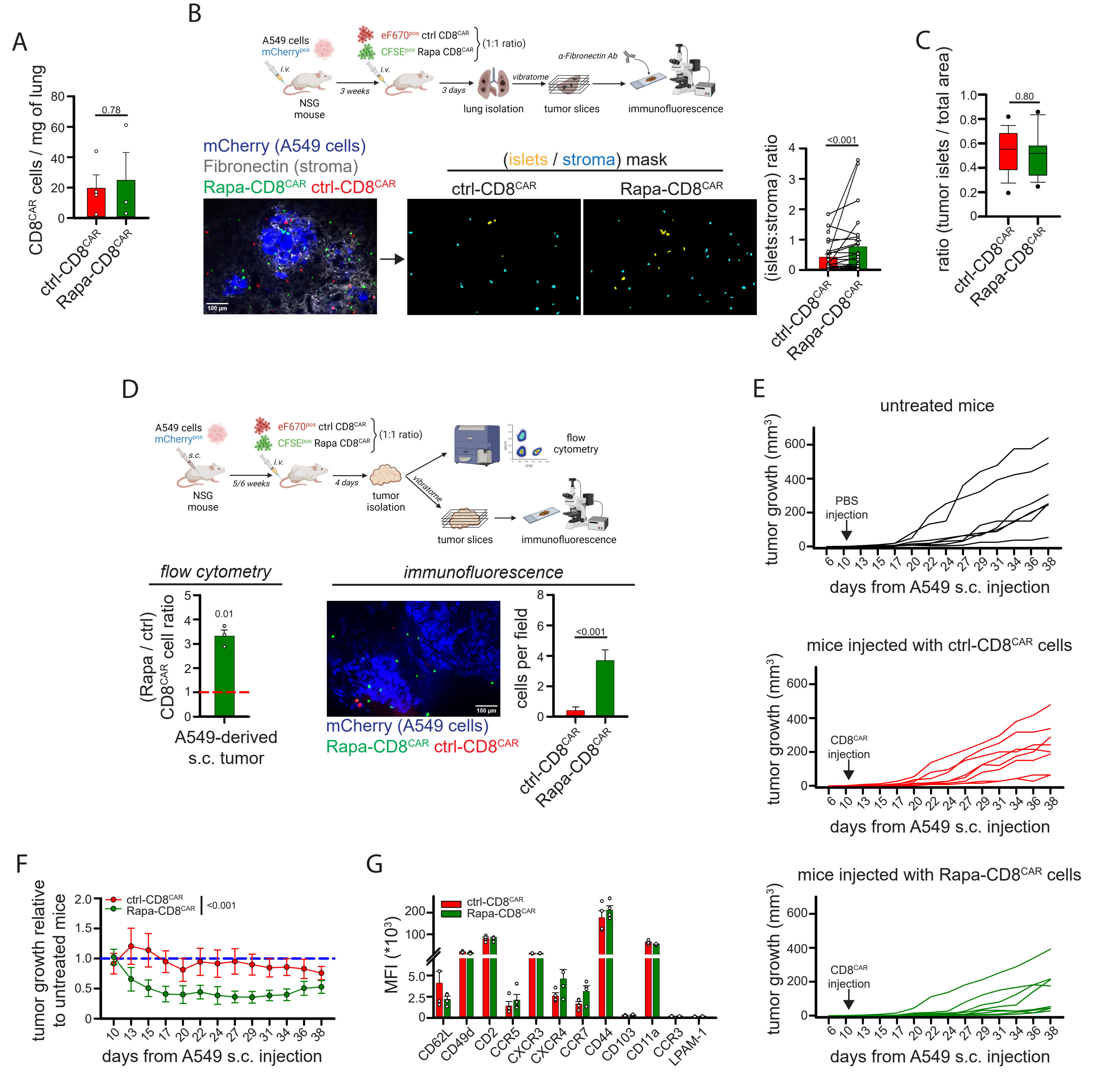

Supplementary Figure 10. Further *in vivo* analyses of the efficacy of Rapa-CD8^{CAR} T cells. Related to Figure 7.
(**A**) Number of anti-EGFR CD8^{+} CAR T cells (per mg of lung) recoverd (flow cytometry) from lung of NSG mice injected i.v. with A549 cells 3-weeks before and injected i.v. with ctrl-CD8^{CAR} or Rapa-CD8^{CAR} cells 4d before (see scheme in figure 7A) (ctrl-CD8^{CAR,} n=4 and Rapa-CD8^{CAR}, n=3 indep. biological samples, unpaired Student‘s T-Test). (**B, D**) eFluor670-labelled ctrl-CD8^{CAR} and CFSE-labelled Rapa-CD8^{CAR} cells have been mixed in a 1:1 ratio and then injected i.v. into NSG mice bearing A549-derived lung tumors (B, as in Fig. 7A) or s.c. tumors (D, as in Fig. 7E). After the indicated time, mice were sacrified and tumors collected for flow cytometry (only for s.c. tumors, n=3 mice, Student‘s T-test vs ctrl) or immunofluorescence analysis (B, n=25 slices, Signed Rank Test; D, n=22 slices, Rank Sum Test). (**C**) Same experiment as in Figure 7E. NSG mice were injected s.c. with with A549 cells and then, 5/6w after, injected i.v. with ctrl-CD8^{CAR} or Rapa-CD8^{CAR} cells. After 4d, mice were sacrificed, and tumors isolated, fixed and cut through vibratome. Here, it is reported the analysis of the relative area of each section occupied by tumor cells (EpCAM^{+}) obtained from immunofluorescence images as shown in Figure 7E (n=18 ctrl and n=14 Rapa microscope fields, unpaired Student‘s T-Test). (**E-F**) Same experiment as in Figure 7F. Tumor growth was measured in NSG mice s.c. injected with A549 cells at indicated days since A549 cell injection. Ctrl-CD8^{CAR} or Rapa-CD8^{CAR} cells was injected i.v. at 10d. In E, the single growth curves of tumors in untreated NSG mice (top) or NSG mice injected with ctrl-CD8^{CAR} (middle) or Rapa-CD8^{CAR} cells (bottom) are reported (untreated, n=7; ctrl-CD8^{CAR,} n=8; Rapa-CD8^{CAR}, n=8 mice). In F (n=8 mice, Rank Sum Test), it is reported the size of tumors growing in NSG mice treated with ctrl-CD8^{CAR} or Rapa-CD8^{CAR} cells relative to uninjected tumor-bearing NSG mice in the same cage (mean tumor size of untreated mice group set to 1 for each cage). (**G**) Surface expression level of the indicated proteins in ctrl-CD8^{CAR} and Rapa-CD8^{CAR} cells (n=4 mice, unpaired Student‘s T-Test). Data are shown as mean ± SEM in A, B and D, F and G, as box plot (center line, median; box limits, upper and lower quartiles; whiskers, 1.5x interquartile range; points, 5th and 95th percentiles) in C, and as raw lines in E. Scale bar 100µm in B and D. Schemes in B and D have been created with BioRender.com.

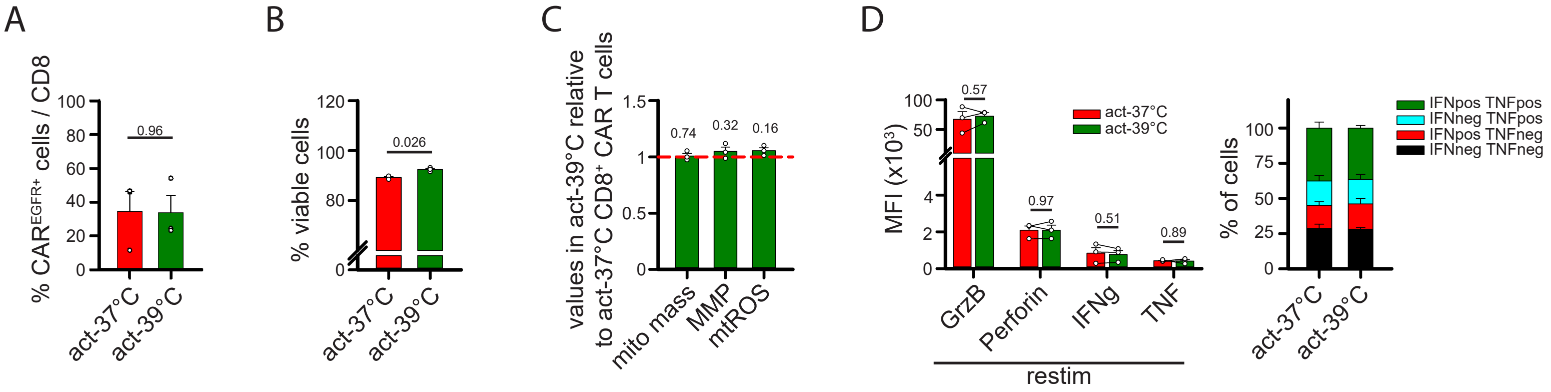

Supplementary Figure 11. Further analyses in $CD8^+$ CAR T cells activated at 37°C or 39°C. Related to Figure 8.
(**A-D**) $CD8^+$ T cells have been activated at 37°C (act-37°C) or at 39°C (act-39°C) for 3d. Then, cells have been put at 37°C and infected with lentiviral particles to generate $CD8^+$ CAR T cells recognizing the EGFR antigen (anti-EGFR $CD8^+$ CAR T cells) and subsequently expanded at 37°C. After 1w, the following parameters were measured: percentage of cells expressing anti-EGFR CAR molecule at the surface (A, n=3 independent biological samples, unpaired Student‘s T-Test), percentage of viable cells (B, n=3 independent biological samples, unpaired Student‘s T-Test), the amount of mitochondria (mito mass: MitoTrackerGreen/FSC ratio in act-39°C relative to act-37°C; C, n=3 independent biological samples, unpaired Student‘s T-Test), the mitochondrial membrane potential (MMP: TMRE/MitoTrackerGreen ratio in act-39°C relative to act-37°C; C, n=3 independent biological samples, unpaired Student‘s T-Test), the amount of mtROS (MitoSox/FSC ratio in act-39°C relative to act-37°C; C, n=3 independent biological samples, unpaired Student‘s T-Test), and the level (MFI) of the indicated protein in restimulated (restim, 5h with human TransAct + BfdA) cells (for IFNg and TNF the percentage of cells producing these cytokines are indicated in the graph on the right) (D, n=3 independent biological samples, unpaired Student‘s T-Test). All data are shown as mean ± SEM.

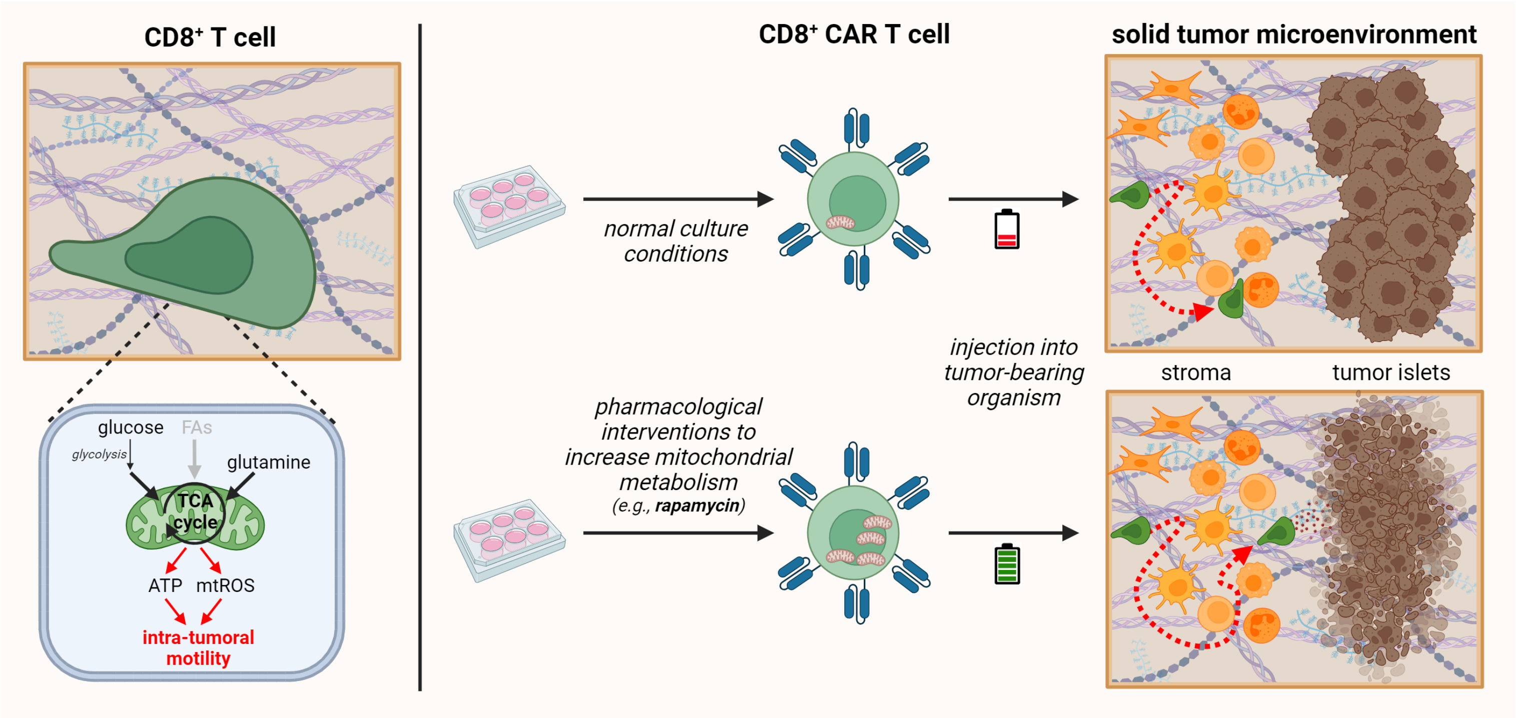

Supplementary Figure 12. Summary of the main findings reported in the manuscript.
On the left, TCA cycle fueled by glucose and glutamine, but not fatty acids (FAs), is the main metabolic pathway supporting human CD8$^+$ T cell migration in 3D environments, including solid tumors. Mechanistically, TCA cycle is required to support both ATP and mtROS production via OXPHOS machinery. On the right, pharmacological strategies used during *in vitro* culture to increase the mitochondrial metabolism of CD8$^+$ CAR T cells are effective to confer on these cells a superior intratumoral motility to reach tumor islets for efficient killing of cancer cells in preclinical models of human solid tumors. Figure created with BioRender.com.

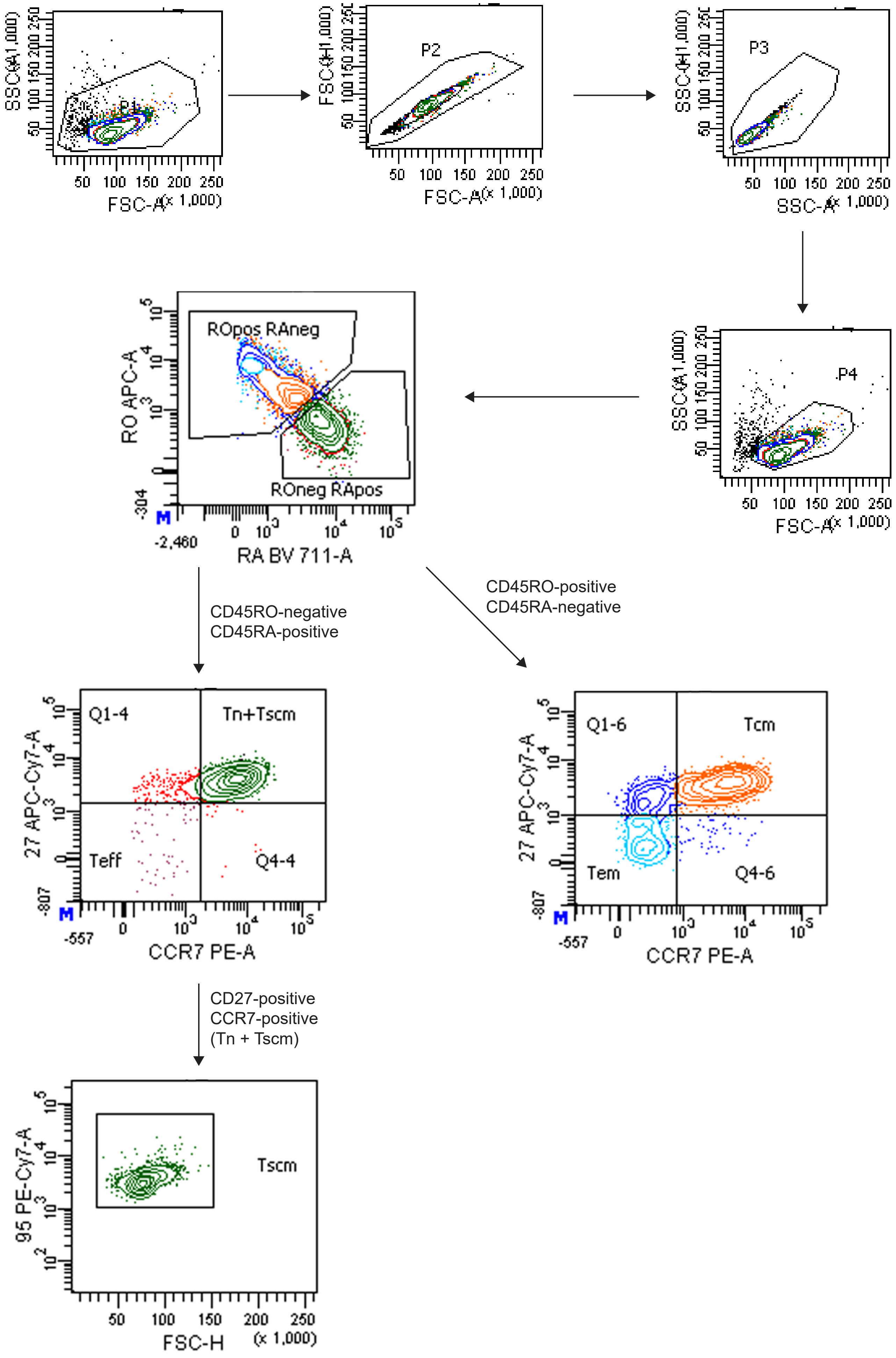

Supplementary Figure 13. Gating Strategy for flow cytometry.
Representative gating strategy to define memory/effector subpopulation in flow cytometry as reported in figure 6F and S6G.

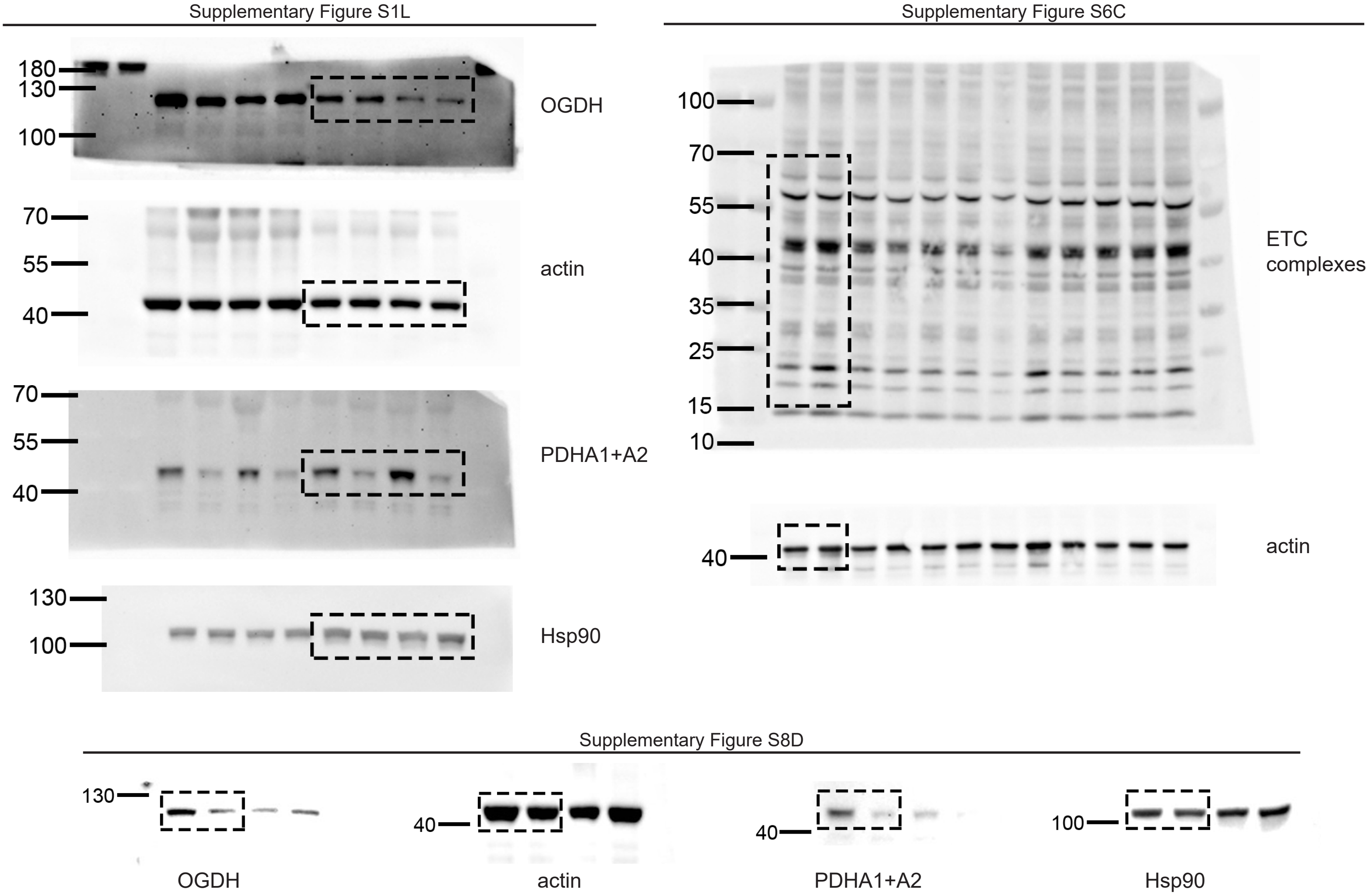

Supplementary Figure 14. Original WB scans.
Raw images of western blot acquisitions used in the manuscript.
