# Supplementary material for: Mitochondrial metabolism sustains CD8+ T cell migration for an efficient infiltration into solid tumors
Source: Nat Commun. 2024 Mar 11;15:2203. doi: 10.1038/s41467-024-46377-7 (PMC10928223; doi:10.1038/s41467-024-46377-7)
Supplement: Supplementary file 3 — Description of Additional Supplementary Files [file 41467_2024_46377_MOESM3_ESM.pdf]

Description of Additional Supplementary Files

Supplementary Movies 1-58. This zip archive contains 58 movies of the 3D migration experiments shown in the main figures (2D z-stack reconstruction, 3 fps).
