# Supplementary material for: Mitochondrial metabolism sustains CD8+ T cell migration for an efficient infiltration into solid tumors
Source: Nat Commun. 2024 Mar 11;15:2203. doi: 10.1038/s41467-024-46377-7 (PMC10928223; doi:10.1038/s41467-024-46377-7)
Supplement: Supplementary file 5 — Reporting Summary [file 41467_2024_46377_MOESM5_ESM.pdf]

nature portfolio

Corresponding author(s): Luca SIMULA
Emmanuel DONNADIEU

Last updated by author(s): Jan 31, 2024

Reporting Summary

Nature Portfolio wishes to improve the reproducibility of the work that we publish. This form provides structure for consistency and transparency in reporting. For further information on Nature Portfolio policies, see our Editorial Policies and the Editorial Policy Checklist.

Statistics

For all statistical analyses, confirm that the following items are present in the figure legend, table legend, main text, or Methods section.

n/a	Confirmed	
☐	☒	The exact sample size (*n*) for each experimental group/condition, given as a discrete number and unit of measurement
☐	☒	A statement on whether measurements were taken from distinct samples or whether the same sample was measured repeatedly
☐	☒	The statistical test(s) used AND whether they are one- or two-sided<br>*Only common tests should be described solely by name; describe more complex techniques in the Methods section.*
☒	☐	A description of all covariates tested
☐	☒	A description of any assumptions or corrections, such as tests of normality and adjustment for multiple comparisons
☐	☒	A full description of the statistical parameters including central tendency (e.g. means) or other basic estimates (e.g. regression coefficient) AND variation (e.g. standard deviation) or associated estimates of uncertainty (e.g. confidence intervals)
☐	☒	For null hypothesis testing, the test statistic (e.g. *F*, *t*, *r*) with confidence intervals, effect sizes, degrees of freedom and *P* value noted<br>*Give P values as exact values whenever suitable.*
☒	☐	For Bayesian analysis, information on the choice of priors and Markov chain Monte Carlo settings
☒	☐	For hierarchical and complex designs, identification of the appropriate level for tests and full reporting of outcomes
☒	☐	Estimates of effect sizes (e.g. Cohen's *d*, Pearson's *r*), indicating how they were calculated

Our web collection on statistics for biologists contains articles on many of the points above.

Software and code

Policy information about availability of computer code

Data collection	Live recording of T cell motility in collagen gels was performed using a Nikon epifluorescence microscope equipped with Metamorph software (version 7.10.2.240, Molecular Devices). Live recording of T cell motility within viable tumor slices was performed using a spinning-disk (Yokogawa CSU-X1) Leica DM6000FS microscope equipped with Metamorph software (version 7.8.9, Molecular Devices).<br>Immunofluorescence images were acquired using a spinning-disk (Yokogawa CSU-W1 T1) Ixplore IX83 microscope (Olympus) equipped with ORCA-Flash 4.0 V3 (sCMOS Hamamatsu) camera and CellSens Dimension software (Olympus, v4.1.1).<br>Cytometry samples were analysed using BD Accuri C6 (CFlow Plus software v1.0.264.15) or BD Fortessa (FACSDive software v6.1.3).<br>Tumor growth in the lungs was followed by bioluminescence using PhotonIMAGER system (Biospacelab) and analysis was performed using M3Vision software (Biospacelab, software version 1.2.1.32016).<br>Western blot images were obtained using Fusion FX instrument (Vilber Lourmat, software version 16.16.0.0).<br>Lactate measurements were performed using CLARIOstar instrument (BMG Labtech, software version 5.6.1).<br>Oxygen consumption rate (OCR) was measured using Oxygraph-2k respirometer (Oroboros).
Data analysis	Motility analyses were performed using the TrackMate plugin (v.7.11.1) available in ImageJ. LoG detector was used to identify spots (quality and maximal intensity filters applied for collagen assays) and Simple LAP Tracker to identify tracks (linking max distance 20µm, closing-gap max distance 20µm, frame gap 3). Only tracks containing 10 or more spots were considered for analysis.

For manuscripts utilizing custom algorithms or software that are central to the research but not yet described in published literature, software must be made available to editors and reviewers. We strongly encourage code deposition in a community repository (e.g. GitHub). See the Nature Portfolio guidelines for submitting code & software for further information.

Data

Policy information about availability of data

All manuscripts must include a data availability statement. This statement should provide the following information, where applicable:

- Accession codes, unique identifiers, or web links for publicly available datasets
- A description of any restrictions on data availability
- For clinical datasets or third party data, please ensure that the statement adheres to our policy

Raw immunofluorescence images of NSCLC samples generated in this study have been deposited on Zenodo with accession number [10.57889] and DOI number (https://doi.org/10.57889/Simula.et.al.2023) with unrestricted access. All other data supporting the findings of this work are available in the main text, the Supplementary Information, and Source Data files. A Reporting Summary for this article is available. “Source Data” are provided within this paper.

Research involving human participants, their data, or biological material

Policy information about studies with human participants or human data. See also policy information about sex, gender (identity/presentation), and sexual orientation and race, ethnicity and racism.

Reporting on sex and gender	Data have been collected anonimously regardless of sex and/or gender (both male and female patients or blood donors were used).
Reporting on race, ethnicity, or other socially relevant groupings	Blood from human donors and biopsies from NSCLC patients were collected independently from any racial, ethnic or social grouping.
Population characteristics	Blood from human donors and biopsies from NSCLC patients were collected from adult individuals. No personal information were collected and used in this study.
Recruitment	All human samples were obtained with informed consensus for research purposes.
Ethics oversight	Peripheral blood samples from adult individual were obtained from EFS (Etablissement Français du Sang) under agreement 18/EFS/030, including informed consensus for research purposes. Blood samples were anonymous and not associated with any personal data (including age, gender or sex). Human lung biopsies from NSCLC adult patients were obtained from the Pathology Service of Cochin Hospital without any personal data (including age, gender or sex). Ethical procedures were approved by CPP Ile de France II (approval number 00001072, August 27, 2012) and INSERM Institutional Review Board (CEEI, IRB00003888, approval number 22/893, March 8, 2022), including informed consensus for research purposes.

Note that full information on the approval of the study protocol must also be provided in the manuscript.

Field-specific reporting

Please select the one below that is the best fit for your research. If you are not sure, read the appropriate sections before making your selection.

☒ Life sciences ☐ Behavioural & social sciences ☐ Ecological, evolutionary & environmental sciences

For a reference copy of the document with all sections, see nature.com/documents/nr-reporting-summary-flat.pdf

Life sciences study design

All studies must disclose on these points even when the disclosure is negative.

Sample size	No optimal sample size calculations for in vivo mice experiments were performed. 8 mice for each experimental condition were used from 2 independent experiments to obtain a robust statistical analysis. All experiments in vitro on human T cells were repeated using at least three/four donors for each experimental conditions to obtain a robust statistical analysis.
Data exclusions	No patients or animals were excluded.
Replication	In vitro experiments were performed by using at least three/four donors, which provided consistent and replicable results.<br>For RT-PCR measurements to quantify mtDNA three technical replicates were performed for each donor (with replicable results).<br>For motility analyses in collagen gels, three different stages were acquired for each donor/experimental conditions with replicable results.<br>In vivo experiments were performed using the number of mice indicated in the figure legends. The data obtained were consistent and within the range of normal variability (no outliers).
Randomization	For in vivo experiments on NSG mice implanted with tumors, mice were randomly allocated to treatment groups.<br>For in vitro and ex vivo data, randomization is not relevant as the different experimental conditions were tested using cells derived from the same patient.
Blinding	Investigator was blinded when acquiring tumor measurement data in mice (in vivo data).<br>For in vitro and ex vivo data, blinding was performed during data analysis.

Reporting for specific materials, systems and methods

We require information from authors about some types of materials, experimental systems and methods used in many studies. Here, indicate whether each material, system or method listed is relevant to your study. If you are not sure if a list item applies to your research, read the appropriate section before selecting a response.

Materials & experimental systems

n/a	Involved in the study
☐	☒ Antibodies
☐	☒ Eukaryotic cell lines
☒	☐ Palaeontology and archaeology
☐	☒ Animals and other organisms
☒	☐ Clinical data
☒	☐ Dual use research of concern
☒	☐ Plants

Methods

n/a	Involved in the study
☒	☐ ChIP-seq
☐	☒ Flow cytometry
☒	☐ MRI-based neuroimaging

Antibodies

Antibodies used

For live imaging of cell motility within viable tumor slices, slices were stained with 200ng/slices of anti-EpCAM (BV421 or PE, clone 9C4, Biolegend 324220 or 324206) and anti-gp38 (eFluor660, clone 8.1.1, eBioscience 50-5381-82 or BV421, Biolegend 127423) Abs. NSCLC biopsies were also stained with 200ng/slices of PE-anti-CD8 Ab (Biolegend 344706, clone SK1) to detect endogenous CD8+ T cells and 200ng/slices of Alexa647-anti-fibronectin Ab (BD Bioscience 563098, clone 10/Fibronectin) to detect stroma.

For immunofluorescence stainings, the following antibodies were used (1:50 dilution for cells or 200ng/slice): anti phospho-Ser-19 myosin Light chain 2 (Cell Signaling 3671), anti-tubulin (Sigma T9026, clone DM1A), phalloidin-Alexa647 (Invitrogen A22287), Alexa555-anti-TOMM20 (Abcam ab221292, clone EPR15581-54), Alexa647-anti-ATP5a (Abcam ab196198, clone EPR13030(B)), PE-anti-IDH2 (Abcam ab212122, clone EPR7577), Alexa488-anti-HK1 (Abcam ab184818, clone EPR10134(B)), Alexa488-anti-PFK2/PFKFB3 (Abcam ab203984, clone EPR12594), Alexa405-anti-GLUT1 (Abcam ab210438, clone EPR3915), DyLight405-anti-G6PD (BioTechne NBP2-22125V, clone 2H7), BV605-anti-EpCAM (Biolegend 324224, clone 9C4), PerCP-Cy5.5-anti-CD8 (Biolegend 344710, clone SK1), Alexa647-anti-CD8 (Biolegend 344726, clone SK1), FITC-anti-CD3 (Biolegend 344804, clone SK7), PE-anti-EpCAM (Biolegend 324206, clone 9C4), rabbit anti-cleaved caspase-3 (Cell Signaling 9664S, clone 5A1E) and Alexa647-anti-rabbit secondary antibody (Thermo Fisher A-21245).

For flow cytometry analyses, the following anti-human antibodies were used for extracellular staining (1:100 dilution): BV711 anti-CD45RA (Biolegend 304138, clone HI100), APC anti-CD45RO (Biolegend 304210, clone UCHL1), PE anti-CCR7 (Biolegend 353204, clone G043H7), APC-Cy7 anti-CD27 (Biolegend 302816, clone O323), PE-Cy7 anti-CD95 (Biolegend 305621, clone DX2), CD49D-PE (Biolegend 304303, clone 9F10), CD62L-PerCP (Biolegend 304824, clone DREG-56), CD2-APC (Miltenyi 130-116-253, clone REA972), CCR5-A488 (Biolegend 359103, clone J418F1), CXCR3-PE (Biolegend 353706, clone G025H7), CXCR4-PerCP (Biolegend 306515, clone 12G5), CD44-A647 (Biolegend 397512, clone C44-Mab5), CD103-APC (Biolegend 350216, clone Ber-ACT8), cd11a-PE/Cy5 (Biolegend 301210, clone HI111), CCR3-PE (Biolegend 310705, clone 5E8), LPAM-1-APC (eBioscience 17-5887-82, clone DATK-32), GLUT1-PE (R&D System FAB1418P, clone 202915).

For flow cytometry analyses, the following anti-human antibodies were used for intracellular staining (1:50 dilution): PE-anti-GrzB (Biolegend 372208, clone QA16A02), PE-Cy7-anti-Perforin (Biolegend 353315, clone B-D48), APC-anti-IFNγ (Biolegend 502511, clone 4S.B3), BV421-anti-TNF (Biolegend 502932, clone Mab11), Alexa647-anti-ATP5a (Abcam ab196198, clone EPR13030(B)), PE-anti-IDH2 (Abcam ab212122, clone EPR7577), Alexa488-anti-HK1 (Abcam ab184818, clone EPR10134(B)), Alexa488-anti-PFK2/PFKFB3 (Abcam ab203984, clone EPR12594), Alexa405-anti-GLUT1 (Abcam ab210438, clone EPR3915), DyLight405-anti-G6PD (BioTechne NBP2-22125V, clone 2H7), anti-MCT1 (BioTechne FAB8275T, clone 882616).

For western blot assay, the following primary anti-human antibodies have been used (1:1000 dilution): anti-actin (Cell Signaling 4970, clone 13E5), anti-OGDH (Sigma Aldrich HPA019514), anti-Hsp90 (Cell Signaling 4877S, clone C45G5), anti-PDHA1+A2 (Cell Signaling 2784S) and OxPhos Human WB Antibody Cocktail (Thermo Fisher 45-8199)

Validation

All antibodies used in this study were purchased from companies, no home-made antibodies were generated. Primary antibodies have been validated by the respective manufacturer, as specified in the antibody webpage of each manufacturer:

anti-EpCAM (BV421, Biolegend 324220, clone 9C4)
https://www.biolegend.com/en-us/products/brilliant-violet-421-anti-human-cd326-epcam-antibody-7549?GroupID=BLG5134

anti-EpCAM (PE, Biolegend 324206, clone 9C4)
https://www.biolegend.com/en-us/products/pe-anti-human-cd326-epcam-antibody-3757

anti-gp38 (eFluor660, eBioscience 50-5381-82, clone 8.1.1)
https://www.thermofisher.com/antibody/product/Podoplanin-Antibody-clone-eBio8-1-1-8-1-1-Monoclonal/50-5381-82

anti-gp38 (BV421, Biolegend 127423, clone 8.1.1)

https://www.biolegend.com/en-us/products/brilliant-violet-421-anti-mouse-podoplanin-antibody-18260

PE-anti-CD8 Ab (Biolegend 344706, clone SK1)
https://www.biolegend.com/en-us/products/pe-anti-human-cd8-antibody-6247

Alexa647-anti-fibronectin Ab (BD Bioscience 563098, clone 10/Fibronectin)
https://www.bdbiosciences.com/en-us/products/reagents/flow-cytometry-reagents/research-reagents/single-color-antibodies-ruo/alexa-fluor-647-mouse-anti-fibronectin.563098

anti phospho-Ser-19 myosin Light chain 2 (Cell Signaling 3671)
https://www.cellsignal.com/products/primary-antibodies/phospho-myosin-light-chain-2-ser19-antibody/3671

anti-tubulin (Sigma T9026, clone DM1A)
https://www.sigmaaldrich.com/US/en/product/sigma/t9026

phalloidin-Alexa647 (Invitrogen A22287)
https://www.thermofisher.com/order/catalog/product/A22287

Alexa555-anti-TOMM20 (Abcam ab221292, clone EPR15581-54)
https://www.abcam.com/en-fr/products/primary-antibodies/alexa-fluor-555-tomm20-antibody-epr15581-54-mitochondrial-marker-ab221292

Alexa647-anti-ATP5a (Abcam ab196198, clone EPR13030(B))
https://www.abcam.com/en-fr/products/primary-antibodies/alexa-fluor-647-atp5a-antibody-epr13030b-ab196198

PE-anti-IDH2 (Abcam ab212122, clone EPR7577)
https://www.abcam.com/en-fr/products/primary-antibodies/pe-idh2-antibody-epr7577-ab212122

Alexa488-anti-HK1 (Abcam ab184818, clone EPR10134(B))
https://www.abcam.com/en-fr/products/primary-antibodies/alexa-fluor-488-hexokinase-1-antibody-epr10134b-mitochondrial-outer-membrane-marker-ab184818

Alexa488-anti-PFK2/PFKFB3 (Abcam ab203984, clone EPR12594)
https://www.abcam.com/en-fr/products/primary-antibodies/alexa-fluor-488-pfkfb3-antibody-epr12594-ab203984

Alexa405-anti-GLUT1 (Abcam ab210438, clone EPR3915)
https://www.abcam.com/en-fr/products/primary-antibodies/alexa-fluor-405-glucose-transporter-glut1-antibody-epr3915-ab210438

DyLight405-anti-G6PD (BioTechne NBP2-22125V, clone 2H7)
https://www.bio-techne.com/p/antibodies/glucose-6-phosphate-dehydrogenase-antibody-2h7_nbp2-22125v

BV605-anti-EpCAM (Biolegend 324224, clone 9C4)
https://www.biolegend.com/en-us/products/brilliant-violet-605-anti-human-cd326-epcam-antibody-8886

PerCP-Cy5.5-anti-CD8 (Biolegend 344710, clone SK1)
https://www.biolegend.com/en-us/products/percp-cyanine5-5-anti-human-cd8-antibody-6389

Alexa647-anti-CD8 (Biolegend 344726, clone SK1)
https://www.biolegend.com/en-us/products/alexa-fluor-647-anti-human-cd8-antibody-9764

FITC-anti-CD3 (Biolegend 344804, clone SK7)
https://www.biolegend.com/en-us/products/fitc-anti-human-cd3-antibody-6427

PE-anti-EpCAM (Biolegend 324206, clone 9C4)
https://www.biolegend.com/en-us/products/pe-anti-human-cd326-epcam-antibody-3757

rabbit anti-cleaved caspase-3 (Cell Signaling 9664S, clone 5A1E)
https://www.cellsignal.com/products/primary-antibodies/cleaved-caspase-3-asp175-5a1e-rabbit-mab/9664

Alexa647-anti-rabbit secondary antibody (Thermo Fisher A-21245).
https://www.thermofisher.com/antibody/product/Goat-anti-Rabbit-IgG-H-L-Highly-Cross-Adsorbed-Secondary-Antibody-Polyclonal/A-21245

BV711 anti-CD45RA (Biolegend 304138, clone HI100)
https://www.biolegend.com/en-us/products/brilliant-violet-711-anti-human-cd45ra-antibody-7937

APC anti-CD45RO (Biolegend 304210, clone UCHL1)
https://www.biolegend.com/en-us/products/apc-anti-human-cd45ro-antibody-856

PE anti-CCR7 (Biolegend 353204, clone G043H7)
https://www.biolegend.com/en-us/products/pe-anti-human-cd197-ccr7-antibody-7498

APC-Cy7 anti-CD27 (Biolegend 302816, clone O323)
https://www.biolegend.com/en-us/products/apc-cyanine7-anti-human-cd27-antibody-3611

PE-Cy7 anti-CD95 (Biolegend 305621, clone DX2)
https://www.biolegend.com/en-us/products/pe-cyanine7-anti-human-cd95-fas-antibody-6495

CD49D-PE (Biolegend 304303, clone 9F10)
https://www.biolegend.com/en-us/products/pe-anti-human-cd49d-antibody-584

CD62L-PerCP (Biolegend 304824, clone DREG-56)
https://www.biolegend.com/en-us/products/percp-cyanine5-5-anti-human-cd62l-antibody-4243

CD2-APC (Miltenyi 130-116-253, clone REA972)
https://www.miltenyibiotec.com/FR-en/products/cd2-antibody-anti-human-reafinity-rea972.html#conjugate=apc:size=30-tests-in-60-ul

CCR5-A488 (Biolegend 359103, clone J418F1)
https://www.biolegend.com/en-us/products/alexa-fluor-488-anti-human-cd195-ccr5-antibody-8715

CXCR3-PE (Biolegend 353706, clone G025H7)
https://www.biolegend.com/en-us/products/pe-anti-human-cd183-cxcr3-antibody-7579

CXCR4-PerCP (Biolegend 306515, clone 12G5)
https://www.biolegend.com/en-us/products/percp-cyanine5-5-anti-human-cd184-cxcr4-antibody-6777

CD44-A647 (Biolegend 397512, clone C44-Mab5)
https://www.biolegend.com/en-us/products/alexa-fluor-647-anti-human-cd44-antibody-19448

CD103-APC (Biolegend 350216, clone Ber-ACT8)
https://www.biolegend.com/en-us/products/apc-anti-human-cd103-integrin-alphae-antibody-9940

cd11a-PE/Cy5 (Biolegend 301210, clone HI111)
https://www.biolegend.com/en-us/products/pe-cyanine5-anti-human-cd11a-antibody-698

CCR3-PE (Biolegend 310705, clone 5E8)
https://www.biolegend.com/en-us/products/pe-anti-human-cd193-ccr3-antibody-1662

LPAM-1-APC (eBioscience 17-5887-82, clone DATK-32)
https://www.thermofisher.com/antibody/product/Integrin-alpha-4-beta-7-LPAM-1-Antibody-clone-DATK32-DATK-32-Monoclonal/17-5887-82

GLUT1-PE (R&D System FAB1418P, clone 202915)
https://www.bio-techne.com/p/antibodies/human-glut1-pe-conjugated-antibody-202915_fab1418p

PE-anti-GrzB (Biolegend 372208, clone QA16A02)
https://www.biolegend.com/en-us/products/pe-anti-human-mouse-granzyme-b-recombinant-antibody-14431

PE-Cy7-anti-Perforin (Biolegend 353315, clone B-D48)
https://www.biolegend.com/en-us/products/pe-cyanine7-anti-human-perforin-antibody-12957

APC-anti-IFNγ (Biolegend 502511, clone 4S.B3)
https://www.biolegend.com/en-us/products/apc-anti-human-ifn-gamma-antibody-1012

BV421-anti-TNF (Biolegend 502932, clone Mab11)
https://www.biolegend.com/en-us/products/brilliant-violet-421-anti-human-tnf-alpha-antibody-7215

anti-MCT1 (BioTechne FAB8275T, clone 882616)
https://www.bio-techne.com/p/antibodies/human-mct1-slc16a1-alexa-fluor-594-conjugated-antibody-882616_fab8275t

anti-actin (Cell Signaling 4970, clone 13E5)
https://www.cellsignal.com/products/primary-antibodies/b-actin-13e5-rabbit-mab/4970

anti-OGDH (Sigma Aldrich HPA019514)
https://www.sigmaaldrich.com/US/en/product/sigma/hpa019514

anti-Hsp90 (Cell Signaling 4877S, clone C45G5)

https://www.cellsignal.com/products/primary-antibodies/hsp90-c45g5-rabbit-mab/4877

anti-PDHA1+A2 (Cell Signaling 2784S)
https://www.cellsignal.com/products/primary-antibodies/pyruvate-dehydrogenase-antibody/2784

OxPhos Human WB Antibody Cocktail (Thermo Fisher 45-8199)
https://www.thermofisher.com/antibody/product/OxPhos-Human-WB-Antibody-clone-Cocktail-Cocktail/45-8199

Eukaryotic cell lines

Policy information about cell lines and Sex and Gender in Research

Cell line source(s)	1. Human BxPC3 pancreatic cancer cells (in-house stock).<br>2. Human A549 lung cancer cells (in-house stock).<br>3. Human HEK-293T cells tumor cells (in-house stock).<br>4. Human HUVEC primary cells (Thermo Fisher C0035C lot number 2492384) from male newborn.
Authentication	Human HUVEC cells were purchased from Thermo Fisher (C0035C).<br>BxPC3, A549 and HEK cell lines were not authenticated (in-house stock).
Mycoplasma contamination	We confirm that all cell lines have routinely tested negative for mycoplasma.
Commonly misidentified lines (See ICLAC register)	No commonly misidentified cell lines were included within the study.

Animals and other research organisms

Policy information about studies involving animals; ARRIVE guidelines recommended for reporting animal research, and Sex and Gender in Research

Laboratory animals	NOD.Cg-Prkdc(scid)Il2rg(tm1Wjll)/SzJ (abbreviated NSG) immunodeficient mice (The Jackson Laboratory, Bar Harbor, ME, USA, 005557) were housed in pathogen-free condition at Cochin Institute Animal Facility. Mice were kept in cages of no more than 5-6 mice each, divided by sex, under 12h/12h light/dark cycles, with standard temperature, humidity and pressure conditions according to FELASA guidelines. Small red squared mice house and paper were used for cage enrichment. Mice health was monitored by veterinary staff and health analysis for pathogens were performed according to FELASA guidelines. All efforts were made to minimize animal suffering and to reduce the number of mice used, in accordance with the European Communities Council Directive of 24 November 1986 (86/609/EEC).
Wild animals	No wild animals were used in this study.
Reporting on sex	Mice were used independently from sex (both females and males). Data and results obtained are not restricted to a specific sex.
Field-collected samples	The study did not involve samples collected from the field.
Ethics oversight	The mice protocol has been approved by Paris Descartes University (CEEA 17-039) and the French Ministry of Research (APAFiS #15076).

Note that full information on the approval of the study protocol must also be provided in the manuscript.

Flow Cytometry

Plots

Confirm that:

- [x] The axis labels state the marker and fluorochrome used (e.g. CD4-FITC).
- [x] The axis scales are clearly visible. Include numbers along axes only for bottom left plot of group (a 'group' is an analysis of identical markers).
- [x] All plots are contour plots with outliers or pseudocolor plots.
- [x] A numerical value for number of cells or percentage (with statistics) is provided.

Methodology

Sample preparation	Antibodies for extracellular proteins were incubated for 20min at RT in DPBS solution containing 0.5% BSA. True-Nuclear Transcription Factor Buffer Set (Biolegend 424401) was used to stain intracellular proteins.
Instrument	Samples were analysed using BD Accuri C6 or BD Fortessa cytometers.
Software	CFlow Plus software and DIVA software were used for data analysis.

Cell population abundance	No cell sorting was performed.
Gating strategy	For T cell subpopulations, the following subsets were defined by flow cytometry gating strategy:<br>-) stem cell memory-like (Tscm) CD45ROnegCD45RAposCD27posCCR7posCD95pos;<br>-) naïve-like (naive) CD45ROnegCD45RAposCD27posCCR7posCD95neg;<br>-) effector-like (Teff) CD45ROnegCD45RAposCD27negCCR7neg;<br>-) central memory-like (Tcm) CD45ROposCD45RAnegCD27posCCR7pos;<br>-) effector memory-like (Tem) CD45ROposCD45RAnegCD27negCCR7neg.<br>Cells outside the indicated gates were defined as “others”. Gating strategy is provided in Supplementary Figure 13.

☒ Tick this box to confirm that a figure exemplifying the gating strategy is provided in the Supplementary Information.
